# Multi-Agent Team Learning in Virtualized Open Radio Access Networks (O-RAN)

**DOI:** 10.3390/s22145375

**Published:** 2022-07-19

**Authors:** Pedro Enrique Iturria-Rivera, Han Zhang, Hao Zhou, Shahram Mollahasani, Melike Erol-Kantarci

**Affiliations:** School of Electrical Engineering and Computer Science, University of Ottawa, Ottawa, ON K1N 6N5, Canada; pitur008@uottawa.ca (P.E.I.-R.); hzhan363@uottawa.ca (H.Z.); hzhou098@uottawa.ca (H.Z.); smollah2@uottawa.ca (S.M.)

**Keywords:** multi-agent systems, team learning, O-RAN, xApps

## Abstract

Starting from the concept of the Cloud Radio Access Network (C-RAN), continuing with the virtual Radio Access Network (vRAN) and most recently with the Open RAN (O-RAN) initiative, Radio Access Network (RAN) architectures have significantly evolved in the past decade. In the last few years, the wireless industry has witnessed a strong trend towards disaggregated, virtualized and open RANs, with numerous tests and deployments worldwide. One unique aspect that motivates this paper is the availability of new opportunities that arise from using machine learning, more specifically multi-agent team learning (MATL), to optimize the RAN in a closed-loop where the complexity of disaggregation and virtualization makes well-known Self-Organized Networking (SON) solutions inadequate. In our view, Multi-Agent Systems (MASs) with MATL can play an essential role in the orchestration of O-RAN controllers, i.e., near-real-time and non-real-time RAN Intelligent Controllers (RIC). In this article, we first provide an overview of the landscape in RAN disaggregation, virtualization and O-RAN, then we present the state-of-the-art research in multi-agent systems and team learning as well as their application to O-RAN. We present a case study for team learning where agents are two distinct xApps: power allocation and radio resource allocation. We demonstrate how team learning can enhance network performance when team learning is used instead of individual learning agents. Finally, we identify challenges and open issues to provide a roadmap for researchers in the area of MATL based O-RAN optimization.

## 1. Introduction

The demand for mobile connectivity has been undeniably growing over the past decades, including a parallel increase in the demand for better Quality of Service (QoS). On top of that, 5G and the next generations of mobile networks will not only serve smart phone users but also verticals such as self-driving cars, healthcare, manufacturing, gaming, marketing, Internet of Things (IoT) and many more [1]. In almost all generations of mobile networks, resource optimization has been a challenge, yet with 5G, despite new spectrum allocations in the mmWave band, the spectrum is still scarce with respect to increasing demand for wireless connectivity due to emerging new applications such as virtual reality, remote surgery, and so on [2]. Moreover, starting from Long-Term Evolution (LTE), the densification trend continues with 5G, at the cost of increased investments. It is well-known that densification is essential due to the limited coverage of mmWave bands. Hence, the increasing complexity of mobile networks is reaching the limits of model-based optimization approaches and yielding to data-driven approaches, also known as AI-enabled wireless networks [3]. In this context, the interest in self-optimizing networks that use machine learning is growing [4]. In the meanwhile, an interesting nexus is emerging from AI-enabled networks and RAN disaggregation, virtualization and open interfaces.

In 2010s, Cloud RAN (C-RAN) introduced a disaggregated architecture that allowed grouping Baseband Units (BBUs) into a centralized BBU pool, with the potential of reducing deployment costs in the long term and improving network capacity. Other architectures have continued from the path of C-RAN, such as virtualized RAN (vRAN) which adds the concept of hardware functions being softwarized. RAN disaggregation and virtualization offer many advantages over traditional RAN solutions, such as reduced network deployment time, improved energy efficiency and enhanced mobility support. Most recently, the Open Radio Access Network (O-RAN) Alliance has brought together those prior efforts around a new RAN architecture with open interfaces, aiming to replace some of the interfaces that have become proprietary over time due to vendor specific implementations of standards. Open interfaces are considered to be important for equipment from multiple vendors to be stacked together and for the selection of best-of-breed solutions.

The timing of O-RAN coincides with new advances in Software Defined Networking, Network Function Virtualization, dynamic function splitting, high capacity data centers and cloud and edge computing, all of which have set the stage for O-RAN [5]. Meanwhile, the increased complexity and the flexible architecture of the O-RAN specifications call for the use of machine learning techniques more than ever.

As of 2021, there are many implementations of open RAN around the globe. For instance, in Japan, Rakuten has deployed distributed data centers hosting both Distributed Unit (DU) and Central Unit (CU) functions. The Spanish operator Telefónica has invested in the integration of its open RAN solution with its multi-access edge computing (MEC) solution. In addition, some operators such as Dish, Sprint and T-Mobile in the US and Etisalat in the Middle East are in the testing phase of open RAN. There are many other emerging vendors, system integrators and operators who are experimenting with open RAN, in addition to the above mentioned players in the market. It is worth noting that, some of these implementations are not necessarily following O-RAN specifications defined by O-RAN Alliance.

The multitude of layers, functions, splits, and the consequent complexity in O-RAN, position machine learning techniques that involve multiple agents and team learning as potential solutions. In recent years, MASs have been used to address complex problems in robotics and other self-driving systems therefore they can be promising alternatives for the self-driving (or self-optimizing) capability of O-RAN. In team learning, distributed agents form teams that cooperate to reach a common goal. The utmost goal of this aggregation is to maximize the team’s objective function. The network function softwarization capabilities of O-RAN present an excellent opportunity to utilize teams of distributed agents as cooperating xApps to improve main key performance indicators while sharing partial or complete information on observations. To the best of our knowledge, this paper is the first to elaborate on multi-agent team learning (MATL) and its use in O-RAN. Note that providing a survey on MAS or description of O-RAN specifications is beyond the scope of this paper. In the literature, there are several comprehensive surveys on MAS [6] and O-RAN specifications [7,8,9].This paper focuses on the application of MATL in O-RAN. We first begin with a background on recent advances in RAN architectures leading to O-RAN, and then we continue with an introduction on MAS and team learning. We discuss a case study where MATL is employed in O-RAN. More specifically, we study three schemes for team learning where each team is comprised by two xApps for optimizing power allocation and resource allocation. Finally, we provide a detailed discussion on the challenges of MATL in O-RAN, and identify open issues in the field.

## 2. Background on Disaggregated, Virtualized RAN and O-RAN

Traditional RAN solutions offer an architecture where BBUs and RUs are co-located. This brought limitations in terms of not being able to pool BBU resources. Therefore the following generation of RAN architectures considered BBU resources that are pooled close to the radios but not co-located, where geographical proximity is necessary due to latency limitations. The pool of BBUs is called as Distributed Unit (DU), and the radios constitute the Radio Unit (RU). Within O-RAN specifications, another level of processors is also defined, which is called as O-RAN Central Unit (O-CU) (In O-RAN specs, the radio unit is called as O-RU, and the distributed computational unit is called as O-DU).

The most appealing reason behind RAN disaggregation was to reduce costs and bring more versatility to the technological market. An earlier version of RAN disaggregation was seen in C-RAN where some hardware functions are implemented as software functions, and BBU functionality is collected at the centralized cloud. C-RAN offers improvements in network capacity, handling cooperative processing, mobility, coverage, energy-efficient network operation, and reduced deployment costs [10].

**Figure 1:**
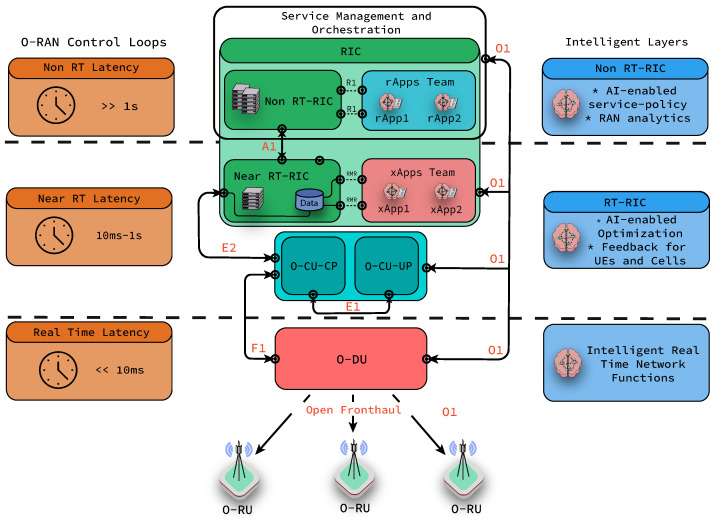
O-RAN interfaces, closed-loop latency requirements and intelligence at different layers of O-RAN. O1 corresponds to the interface between the SMO and O-RAN managed elements. A1 interfaces the SMO and RAN. E2 interfaces the control and optimization of the O-CU and O-DU nodes (E2 nodes) resources by the Near RT-RIC. F1 interfaces the O-DU and O-CU. E1 interface interconnects O-CU-CP and O-CU-UP. The Open Fronthaul refers to the interface that connects the O-RU with the O-DU. R1 interfaces rApps and the Non-RT RIC. Finally, xApps are capable to access to E2 nodes’ realtime information and general purpose data in a central database using a service bus implemented by a peer-to-peer low latency (∼0.02 ms) library named RIC Message Router (RMR) [18].

On the evolution path of RAN architectures, the most recent development comes with O-RAN, in which interfaces between O-RU and O-DU and between O-DU and O-CU are based on open specifications [11]. This paves the way to interoperability between vendor products and the possibility to select the best set of products by Mobile Network Operators (MNOs). In addition, O-RAN embraces intelligence in every layer of its architecture and aims to leverage new machine learning-based technologies [12]. As seen in Figure 1, the RAN Intelligent Controller (RIC) is composed of the non-Real Time RIC (non RT-RIC) and the near-Real Time RIC (near RT-RIC), which have been considered fundamental platforms to enable intelligence [13]. The primary objective of the non RT-RIC is to support applications that operate in non-real-time or above the one second time frame. In the non-RT RIC, we employ the rApps that aim at non-real-time parameter optimization, RAN analytics and management, as well as model training for the lower layer of the RIC. On the other hand, the near RT-RIC supports applications that are executed in near-real-time, such as between 10ms and 1ms. The near-RT RIC has two interfaces with a centralized unit user plane (O-CU-UP) and control plane (O-CU-CP) used for data transmission and signaling, and configuration, respectively. In the case of near RT-RIC, xApps enable intelligence by enforcing policies from the non RT-RIC and are capable but not restricted to executing Radio Resource Management (RRM) tasks such as cell load prediction, anomaly detection, traffic steering and data collection from E2 Nodes (O-DU, O-CU and O-RU). Access from the near RT-RIC and non RT-RIC to xApps and rApps is provided through Application Programming Interfaces (APIs) with software updates and data gathering purposes. The introduction of xApps and rApps increase the interoperability and the openness of the O-RAN architecture by allowing developers from third-party software providers to share, publish and provide access to their applications. As a last remark, it can be seen in the above-mentioned figure that the distributed unit (O-DU) is connected to O-CU, and through O-RUs, it can provide services to UEs.

### The Role of Intelligence in O-RAN

O-RAN defines certain guidelines to employ AI in its architecture. Offline and online learning is expected to coexist with a modular design as best practice to follow. This will enable service providers to decide the location of intelligence in the network functions according to their best interests. As a recommendation, xApps and rApps are expected to fall in certain control loops [14,15] according to the time budget needed for such applications (see Figure 1). Furthermore, open-source solutions, such as Acumos AI, emerge as a potential development platform for ML models. Several AI/ML use cases are already identified by the O-RAN community, such as QoE (Quality of Experience) optimization, traffic steering [16], user access control [17] and V2X handover management [12]. It is important to highlight that although some ML applications are described in O-RAN reports, the huge potential of applying MATL in O-RAN are not covered in any of the use cases. On the other hand, MAS has been studied in the context of wireless networks in a few studies. Most of these prior works consider multiple uncoordinated agents working within the same environment, driven by the same reward or goal. However, when multiple agents interact with the environment independently, they change the environment of each other. Additionally, when agents have different goals, the problem cannot be simplified to deploying independent agents. Therefore, using teams of agents in O-RAN emerges as a promising approach.

In Section 4 we present a case study where a team of two xApps exchange information to enhance performance and avoid conflict among team members. Note that, the organization of xApps in teams will incur communication overhead. Before we elaborate more on such opportunities and challenges, in the following section we provide background on MAS and team learning.

## 3. Multi-Agent Systems and Team Learning

MAS is composed of a group of autonomous and intelligent agents that act in an environment in order to accomplish a common goal or individual goals. In the following, we introduce different types of MAS, team learning in MAS, and finally how MAS with team learning can be employed in O-RAN architecture.

A recent survey on open virtualized networks [19], gives a comprehensive overview of the state-of-the-art in modern RANs. In the next section, we summarize the efforts in the intersection of AI and O-RAN.

### 3.1. Types of MAS

There can be several types of MAS, such as homogeneous/heterogeneous, communicating/non-communicating and cooperative (collaborative)/competitive.

**Homogeneous/Heterogeneous**: The homogeneous multi-agent system consists of agents with a similar internal structure, which means that all agents have the same local goals, capabilities, actions, and inference models. In the homogeneous architecture, the main difference among agents is based on the place where their actions are applied over the environment. By contrast, in the heterogeneous MAS, agents may have different goals, capabilities, actions, and inference models. From the O-RAN perspective, homogeneous agents would be instances of the same xApp instantiated for different slices, while different xApps would be heterogeneous agents.

**Communicating/non-communicating:** A group of agents can be designed to communicate with each other or not. When there is no communication, agents act independently without receiving any feedback from other agents. However, since they are working in the same environment, indirect feedback on the actions of the other agents will be observed by the individual agent. For the case of communicating agents, there is explicit feedback among agents. This is more suitable for many O-RAN ML algorithms. Note that the way agents communicate will impact the required bandwidth on the O-RAN interfaces. It is also important to note that in a multi-vendor environment, some agents controlling different vendor functions might not communicate with other.

**Cooperative/Competitive**: In an MAS, agents can be cooperative, competitive, or mixed. In a cooperative setting, the agents need to take collaborative actions to achieve a shared goal, or in other words, agents must jointly optimize a single reward signal. On the other hand, in a competitive setting, each agent tries to maximize its received reward under the worst-case assumption; meanwhile, the other agents always try to minimize the reward of others. It is also possible to have groups with mixed behavior agents where some are cooperative while others are competitive. In the O-RAN architecture, this kind of behavioral differences may be needed for various use cases, where certain xApps might be cooperating while others are competing due to the nature of shared resources. Although many machine learning techniques have been considered for MAS, team learning could have particular significance in the future since it can have applications in O-RAN by organizing xApps as teams of teams in hierarchies. In the next section, we focus on MATL and its potential use in O-RAN.

### 3.2. Multi-Agent Team Learning

**Figure 2:**
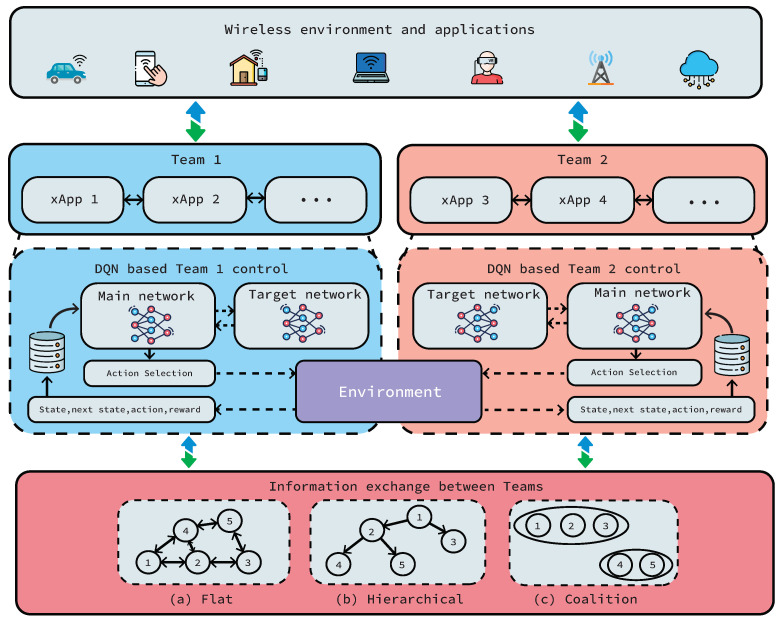
Multi-agent team learning: The figure shows two teams comprised of more than one xApp where each xApp is a DQN agent that interacts with the environment. Teams can form different communication structures to share information: (**a**) flat, (**b**) hierarchical and (**c**) coalition. In our case study, we consider the interactions of one team of agents.

As shown in Figure 2, in team learning, teams of distributed agents cooperate to achieve a common goal. They usually share complete information on observations. More specifically, team learning tries to maximize a team’s objective function comprised of its agents’ contributions. Consequently, this learning approach could be useful in O-RAN architecture where a team of xApps (scheduler, power adaptation, beamforming, etc.) needs to find the best arrangement for their members to improve the overall network performance. Such tasks could be comprised of several individual subtasks corresponding to each team member. Hence, team learning shows itself as a promising approach for the decentralized and granular architecture of O-RAN. Although the application of team learning to O-RAN is new, team learning has been studied and applied for a wide range of applications. For instance, team learning has been used to address complex problems; from multi-robot teams (e.g., robot soccer), multi-player games (e.g., Dota, StarCraft), predator–prey pursuit and capture problems, to search and rescue missions, as well as mitigating traffic congestion.

For instance, Ref. [20] presents an interesting work related to team learning in urban network traffic congestion. The authors proposed a Dynamic Traffic Assignment (DTA) algorithm based on a collaborative, decentralized heterogeneous reinforcement learning approach to mitigate the effects of the randomness of urban traffic scenarios. To this end, two-agent teams are defined as advisers and deciders. The decider team task consist of assigning traffic flows to a specific traffic network. Meanwhile, the adviser team is responsible for supporting the deciders with additional information on the environment.

In particular, the adviser–decider type of team learning can be used in O-RAN considering the different time granularities of non-real-time RIC (non-RT RIC) and near-Real-Time RIC (near-RT RIC). For instance, a team of agents at non-RT RIC can advise another team at near-RT RIC on long-term policies. In the next section, we explore more on how MATL can be useful for O-RAN.

Finally, it is also important to highlight the differences between team learning and distributed or federated learning. Federated learning indicates how the data are used to train/update local models using the information from heterogeneous/homogeneous agents [21] and does not cover the specific dynamics of agent interactions such as in a team. Team learning is not constrained to a specific data model; agents learn by interacting with the environment and they converge on a policy.

### 3.3. Embedding Intelligence in O-RAN

Multi-agent team learning presents itself as an exciting technique where agents can join forces to achieve a common goal. Modular disaggregation and feedback availability throughout the O-RAN architecture intuitively leads us to the applicability and integration of team learning in O-RAN. In order to integrate MATL into O-RAN and develop an AI-enabled RIC, some steps need to be taken.

In the first step, model capabilities and its required resources, including the accessibility of data (observation level), availability of processing power, interaction time, and maximum tolerable latency (processing time and communication time), need to be examined. This evaluation can be executed by the service management and orchestration (SMO) platform. The corresponding RAN counters from O-CUs and O-DUs are gathered in the data collector, which is placed in the SMO. Based on the SMO platform type, various entities can perform the data collection. For instance, if an open network automation platform (ONAP) is employed to play the role of SMO, the virtual event streaming (VES), which is a subsystem in the ONAP will take care of collecting data collection, analytics, and events (DCAE). After collecting analytics, the SMO shares the collected data through a data bus with non-RT RIC. Selecting a proper model and training the model is the next step, where AI agents run and train a model at non-RT RIC, near-RIC, or even O-DU based on the NF resource and latency requirements.

Based on the model’s outcome, agents apply the corresponding actions. Depending on the AI model, type of actions, and location in O-RAN architecture, various interfaces, including A1, O1 and E2, can be used. A1 interface can be used for a policy-based data format, ML/AI model management, and delivering information to the near-RT RIC to apply RAN optimization. Similarly, the E2 interface is located between O-CU, O-DU, and near-RT RIC. Additionally, the O1 interface is responsible for delivering management and operational information of O-DU, O-CU, near-RT RIC and radio unit (O-RU), including performance, configuration and fault management, to the SMO. It can also be used for the configuration of O-DU, O-CU, near-RT RIC and O-RU with respect to the application.

When intelligence is implemented in O-RAN, using a team learning approach, it is important to select a common goal and the sub-goals properly. In addition, the overhead of sharing feedback needs to be considered. In the next section, we present a study case showing the advantages of MATL in the O-RAN architecture.

## 4. A Case Study on Multi-Agent Team Learning in O-RAN

As a case study, we investigate the advantages of using MATL for optimizing resource block and power allocation in O-RAN. Prior studies have shown reinforcement learning based resource block and power allocation outperform traditional approaches [22]. Therefore, we focus on only machine learning approaches in this paper and extend them with the notion of team learning. In our case study, for simplicity, we consider a team of two heterogeneous xApps: one optimizing power allocation and another xApp optimizing resource block allocation.

**Figure 3:**
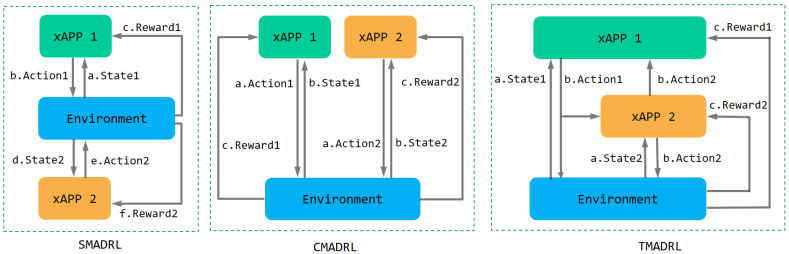
Sequential multi-agent deep reinforcement learning (SMADRL), Concurrent multi-agent deep reinforcement learning (CMADRL) and Team multi-agent deep reinforcement learning (TMADRL) schemes.

The core idea is to let xApps take into account the actions of other team members. This is achieved by changing the state of the xApps to a combination of both environmental information and action information from other team members. In addition, xApps also inform other team members about the actions they decide to take. By exchanging information, team members consider the actions of the others and hence improve the team member’s coordination as well as the overall performance of the RAN. We define our performance metrics as system throughput and energy efficiency. As shown in Figure 3, we propose three schemes to study the interactions among agents and the repercussions of exchanging information. We define our schemes as follows:

- **SMADRL**: In the sequential multi-agent deep reinforcement learning (SMADRL), team members act in a sequential fashion. xApp 1 first observes the environment and takes actions, then xApp 2 acts;
- **CMADRL**: In the concurrent multi-agent deep reinforcement learning (CMADRL), team members act concurrently, allowing in such arrangement, the simultaneous observation and the reward gathering for both xApps;
- **TMADRL**: In the team multi-agent deep reinforcement learning (TMADRL), team members exchange the action information with each other, and make their own decisions accordingly. Specifically, xApp 1 and xApp 2 will take their actions in each time slot based on the team member’s previous action and the environmental states. After sharing such feedback in terms of actions, the agents apply their actions to the system. Finally, they update their models with a single team reward.

### 4.1. Team Learning xApps Markov Decision Process (MDP)

In our case study, both xApps are implemented using deep Q-learning (DQL) [23]. The MDP of two the xApps in the proposed TMADRL scheme are defined as follows:

**1. Power allocation xApp**:

- State space is defined as:
  (1) $$\begin{array}{c} S_{t}^{m}=\{\Gamma_{t}^{n,m^{\prime }},R_{t}^{n,m^{\prime }},p_{t}^{n,m^{\prime }},\\ \;\Gamma_{t}^{n^{\prime },m^{\prime }},R_{t}^{n^{\prime },m^{\prime }},p_{t}^{n^{\prime },m^{\prime }},L_{t}^{n^{\prime },m^{\prime }}\left|n\in N\right\}, \end{array}$$
  where $\Gamma_{t}^{n,m}$ denotes the signal to interference plus noise ratio (SINR) of the transmission link of the *m*th RBG of the *n*th base station (BS), $R_{t}^{n,m}$ denotes the current transmission rate and $p_{t}^{n,m}$ denotes the transmission power. The subscript *t* indicates the time step and $L_{t}^{n,m}$ is the length of queued data in the buffer.

Additionally, $m^{\prime }$, $n^{\prime }$ are respectively the index of resource block that will be allocated to the *k*th user and the index of BS that the *k*th user will be associated in the next time slot. This is given as:

(2) $$\begin{array}{cc} \; & m^{\prime },n^{\prime }\in \\ & \{m^{\prime }\in M,n^{\prime }\in N,\alpha_{t+1}^{n^{\prime },m^{\prime },k}=1|k\in \left\{\alpha_{t}^{n,m,k}=1\right\}\}, \end{array}$$

where $\alpha_{t}^{n,m,k}$ is a binary indicator to indicate whether the *m*th RBG of the *n*th BS is allocated to the *k*th user equipment. *M* corresponds to the number of RBGs of a BS and *N* corresponds to the number of BSs in the environment. The binary RBG allocation indicator of the next time slot is indicated by the action of the resource block allocation xApp. In this way, the chosen action of resource block allocation xApp will be included in the state of power allocation xApp.

- Action space is defined as:

(3) $$A_{t}^{n,m}=\left\{P_{min},P_{min}+\frac{P_{max}-P_{min}}{B-1},\ldots ,P_{max}\right\}.$$

The transmission power values are discretized into *B* levels according to the maximum and the minimum defined values to be assigned to each RBG.

- Reward: The reward is defined as the total throughput minus the sum transmission power, which is given as:

(4) $$r_{t}=\Sigma_{m\in M}\Sigma_{n\in N}\left(R_{t}^{n,m}-\beta P_{t}^{n,m}\right),$$

where β is a factor used to balance the reward of throughput and transmission efficiency.

**2. Radio Resource block allocation xApp**:

- State space is given as:

(5) $$\begin{array}{c} S_{t}^{n}=\{\alpha_{t}^{n,m,k}\Gamma_{t}^{n,m},\alpha_{t}^{n,m,k}R_{t}^{n,m},\alpha_{t}^{n,m,k}p_{t}^{n,m},\\ \alpha_{t}^{n,m,k}L_{t}^{n,m},p_{t+1}^{n,m},\left|m\in M,k\in K\right\}, \end{array}$$

where $p_{t+1}^{n,m}$ is obtained from the power allocation xApp.

- Action space is defined as:

The action of BS *n* is to choose a user by:

(6) $$\begin{array}{cc} A_{t}^{n,m} & =\{k_{0},k_{1},\ldots ,k_{D-1}|k_{d}\in H_{t}^{n}\}, \end{array}$$

where $H_{t}^{n}$ denotes the set of all the user equipment attached to the *n*th BS.

- Reward: The reward of resource block allocation xApp is defined likewise the power allocation xApp.

Both xApps learn and act in the same system with four BSs and 30 user equipments. We use a self-made Python network environment that includes the communication models needed to simulate a mobile network. Finally, the deep reinforcement learning algorithm is implemented with the TensorFlow library. We set up and train the xApps models independently as it is typically performed in the Near RT-RIC of the O-RAN architecture. The simulation settings are shown in Table 1.

### 4.2. Performance Evaluation

**Figure 4:**
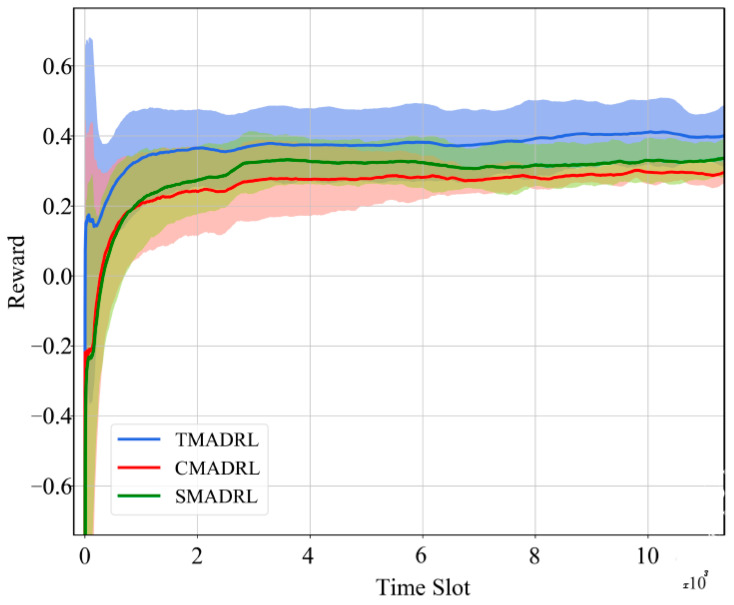
Learning convergence for the TMADRL, CMADRL and SMADRL schemes.

**Figure 5:**
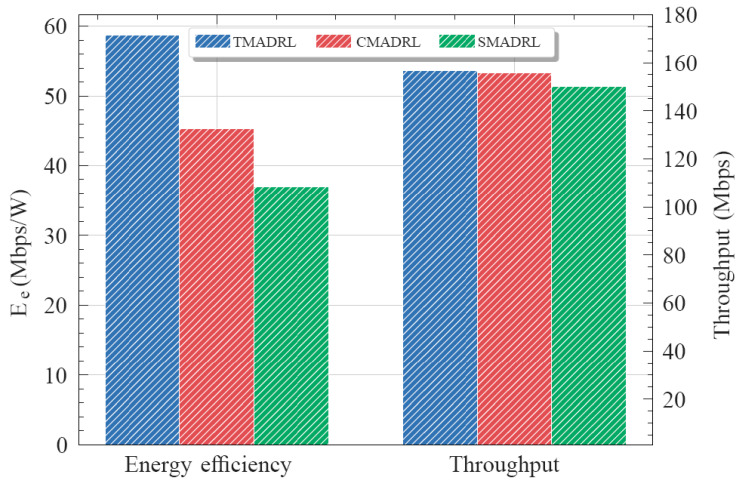
Average energy efficiency ($E_{e}$) and throughput performance metrics for the TMADRL, CMADRL and SMADRL schemes.

**Figure 6:**
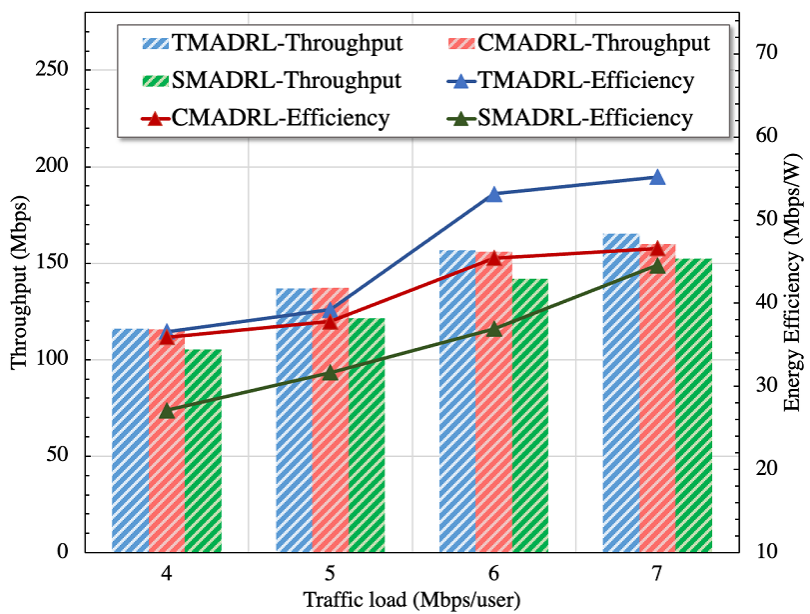
Average energy efficiency ($E_{e}$) and throughput performance metrics for the TMADRL, CMADRL and CMADRL schemes for 4, 5, 6 and 7 Mbps traffic load per user.

Figure 4 shows the convergence curves of the three different algorithms for five trials. It can be observed that the TMADRL algorithm receives a higher reward from the environment compared with the two baselines. Furthermore, in Figure 5, we present the energy efficiency and throughput results of the three schemes under study. The figure indicates that TMADRL achieves a more efficient energy utilization with a gain of 29.7% and 59.04% when compared with CMADRL and SMADRL. Additionally, it can be seen that TMADRL can achieve an improvement of throughput with a gain of 1% and 4.56%. It is important to mention that despite the throughput improvement is not relevant, TMADRL achieves a significant improvement in terms of energy efficiency while maintaining throughput rates. The throughput is measured by the averaged amount of useful data rate per unit of time and the energy efficiency is defined as the ratio of the throughput and the power consumed by the BSs. The reason behind this performance enhancement is related to the capabilities of our team members to exchange information in terms of actions which avoids non-Markovian environment behavior and thus, allowing team convergence. Lastly, in Figure 6, we present four simulation results, each one representing the MATL and baseline behavior under four different traffic loads per user. We can observe a clear positive trend in terms of throughput for the presented algorithms, TMADRL being the best performer among all. Furthermore, an interesting uptrend behavior is observed in the energy efficiency metric that has its roots in the user traffic and the BS resource capacity. When a low user traffic is employed, the transmission capacity is underutilized, meanwhile when the traffic increases, the BS is capable of a more efficient utilization of the resources. Under all the traffic loads, the TMADRL scheme outperforms the baselines presented in this work with a more evident gain in higher loads such as 6 and 7 Mbps. To sum up, MATL shows promising results in the O-RAN architecture with additional improvements when feedback exchange is considered. Note that, despite our use cases focus on xApps, the employed MATL schemes and techniques are extensible to rApps as well. It is also worth mentioning that, under this cooperative setting, the overhead and required processing memory will grow as a trade-off of the increased throughput and efficiency. Specifically, the processing memory will rise by an order of 2N and the communication cost between the two xApps will increase by an order of $2^{N}$, where *N* corresponds to the number of xApps.

In the next section, we elaborate on open issues, as well as the opportunities of MATL in O-RAN.

## 5. Open Issues and Future Directions for Multi-Agent Team Learning in O-RAN

Despite many potential benefits of MATL in O-RAN, several challenges need to be addressed before it can be dominantly used in O-RAN control and optimization. In this section, we identify the challenges, open issues, and opportunities in MATL for O-RAN. It should be noted that some challenges are already fundamental in learning in the MAS environment, while others are only specific to O-RAN.

- **Convergence:** Similar to all other machine learning-based techniques in wireless, MATL for the O-RAN should converge fast. Divergent behavior will cause instability and slow convergence might hinder decision capability in real-time. However, convergence guarantee in decentralized learning for stochastic teams is known to be a challenging problem [24]. For fast convergence, boot strapping techniques or offline training can be considered.
- **Scalability:** Dimensionality issues have been a recurrent issue when the number of agents (so as the states and actions) in an MAS tend to increase. As more xApps become intelligent agents in O-RAN, the scalability of learning, inter-agent communication, and environment feedback need to be addressed.
- **Lack of full observability:** As xApps act in an environment, they will be simultaneously modifying the environment of other agents. Therefore, to take an optimal action, each agent will need to predict other agents’ actions unless all agents can fully observe the environment, which is unlikely in O-RAN. Hence, decisions need to be made based on the agents’ partial observations. This can result in a sub-optimal solution. To this end, learning models that are robust under partial observability need to be explored.
- **Information sharing:** It is essential to decide how much of the available local information should be shared among agents for enhancing the modeling of an environment. This should be jointly considered with fronthaul and midhaul capacity and the functional split decisions in O-RAN.
- **Selecting the optimal team size:** In MATL, choosing the optimal team size can affect learning and system performance. Although a larger team can provide more comprehensive visibility over the environment and access more relevant information, each agent’s incorporation and learning experience can be affected. Meanwhile, one can obtain faster learning within a smaller team size, but a sub-optimal performance may be achieved due to the limited system view. Therefore, optimal team organization will be fundamental to MATL in O-RAN.
- **Goal selection:** In the O-RAN environment, conflicting actions of agents may degrade the network performance, even though each agent intends to maximize the performance. The goal of MATL should be minimizing conflicts and focusing on overall performance enhancement.
- **Impact of delayed feedback and jitter:** Most MAS studies consider that the feedback from the environment is immediate, and if agents are communicating, their feedback is instant. However, in disaggregated RANs, feedback can be delayed. Delayed feedback may cause agents to interact with different observations of the environment and lead to degraded RAN performance.
- **Security and trust:** Most MAS rely on the truthfulness of information shared among agents. Although there are studies on uncertainty or partial observability, intentional wrong reporting and adversarial behavior should also be addressed. This is particularly important when embedding intelligence to O-RAN.

## 6. Conclusions

In this paper, we discuss the potential of using MATL to organize intelligence in O-RAN. We first provide an overview of RAN evolution from C-RAN to vRAN to O-RAN, and then we provide a short summary of MAS and MATL and their applications in O-RAN. Additionally, we present a case study on team learning in the O-RAN architecture by comparing three schemes—sequential multi-agent deep reinforcement learning (SMADRL), concurrent multi-agent deep reinforcement learning (CMADRL) and team multi-agent deep reinforcement learning (TMADRL). Simulation results show that team learning via the TMADRL scheme offers a gain in terms of energy utilization of 29.7% and 59.04% when compared to SMADRL and CMADRL, respectively. Additionally, TMADRL overperforms CMADRL and SMADRL in terms of throughput with a gain of 1% and 4.56%, respectively. Finally, we provide a detailed discussion of the challenges, open issues, and future directions for MATL in O-RAN.

## Figures and Tables

**Table 1:** Simulation settings.

Parameter		Value
Network settings	Networking Environment	4 Base Stations with 1 kilometre inter-site distance, 30 user equipments.
	Propagation	120.9 + 37.6 log10(distance) dB, Log-Normal shadowing: 8 dB.
	Carrier configuration	20 MHz bandwidth, 100 resource blocks, 12 subcarriers per resource block, 12 resource blcok groups (RBGs).
	PHY configuration	Maximum transmission power of 38 dBm, minimum transmission power of 1 dBm, Additive white Gaussian noise = −114 dBm.
	Traffic model	Poisson distribution with varying load between 4–7 Mbps.
	Simulation time	20,000 time slots. 1 time slot is 100 ms.
Machine learning settings	Network structure	Number of hidden layers: 2
	Number of neurons	xApp 1: $N_{h}^{1}=256$ and $N_{h}^{2}=128$, xApp 2: $N_{h}^{1}=512$ and $N_{h}^{2}=256$.
	Discount factor	γ=0.2
	Learning rate	$\alpha_{l}=0.001$
	Reward importance ratio	β=0.35

## Data Availability

Not applicable.

## References

[1] Trakadas P., Sarakis L., Giannopoulos A., Spantideas S., Capsalis N., Gkonis P., Karkazis P., Rigazzi G., Antonopoulos A., Cambeiro M.A. (2021). A Cost-Efficient 5G Non-Public Network Architectural Approach: Key Concepts and Enablers, Building Blocks and Potential Use Cases. Sensors.

[2] Yang P., Kong L., Chen G. (2021). Spectrum Sharing for 5G/6G URLLC: Research Frontiers and Standards. IEEE Commun. Mag..

[3] Elsayed M., Erol-Kantarci M. (2019). AI-Enabled Future Wireless Networks: Challenges, Opportunities, and Open Issues. IEEE Veh. Technol. Mag..

[4] Wang J., Jiang C., Zhang H., Ren Y., Chen K.C., Hanzo L. (2020). Thirty Years of Machine Learning: The Road to Pareto-Optimal Wireless Networks. IEEE Commun. Surv. Tutor..

[5] Chih-Lin I., Kuklinski S., Chen T., Ladid L.L. (2020). A perspective of O-RAN integration with MEC, SON, and network slicing in the 5G era. IEEE Netw..

[6] Dorri A., Kanhere S.S., Jurdak R. (2018). Multi-Agent Systems: A Survey. IEEE Access.

[7] O-RAN Working Group 1 (2022). O-RAN Architecture Description 6.00,” O-RAN.WG1.O-RAN-Architecture-Description-v06.00. https://orandownloadsweb.azurewebsites.net/specifications.

[8] Polese M., Bonati L., D’Oro S., Basagni S., Melodia T. (2022). Understanding O-RAN: Architecture, Interfaces, Algorithms, Security, and Research Challenges. arXiv.

[9] Giannopoulos A., Spantideas S., Kapsalis N., Gkonis P., Sarakis L., Capsalis C., Vecchio M., Trakadas P. (2022). Supporting Intelligence in Disaggregated Open Radio Access Networks: Architectural Principles, AI/ML Workflow, and Use Cases. IEEE Access.

[10] Checko A., Christiansen H.L., Yan Y., Scolari L., Kardaras G., Berger M.S., Dittmann L. (2015). Cloud RAN for Mobile Networks—A Technology Overview. IEEE Commun. Surv. Tutor..

[11] Garcia-Saavedra A., Costa-Perez X. (2021). O-RAN: Disrupting the Virtualized RAN Ecosystem. IEEE Commun. Stand. Mag..

[12] O-RAN Working Group 2 (2021). O-RAN AI/ML Workflow Description and Requirements–v1.03. https://orandownloadsweb.azurewebsites.net/specifications.

[13] Balasubramanian B., Daniels E.S., Hiltunen M., Jana R., Joshi K., Sivaraj R., Tran T.X., Wang C. (2021). RIC: A RAN Intelligent Controller Platform for AI-Enabled Cellular Networks. IEEE Internet Comput..

[14] Bonati L., D’Oro S., Polese M., Basagni S., Melodia T. (2021). Intelligence and Learning in O-RAN for Data-Driven NextG Cellular Networks. IEEE Commun. Mag..

[15] Dryjański M., Kliks A. (2022). The O-RAN Whitepaper 2022 RAN Intelligent Controller, xApps and rApps. https://rimedolabs.com/blog/the-oran-whitepaper-2022-ran-intelligent-controller.

[16] Dryjański M., Kułacz L., Kliks A. (2021). Toward Modular and Flexible Open RAN Implementations in 6G Networks: Traffic Steering Use Case and O-RAN xApps. Sensors.

[17] Cao Y., Lien S.Y., Liang Y.C., Chen K.C. Federated Deep Reinforcement Learning for User Access Control in Open Radio Access Networks. Proceedings of the IEEE International Conference on Communications.

[18] O-RAN-SC RIC Message Router–RMR. https://docs.o-ran-sc.org/projects/o-ran-sc-ric-plt-lib-rmr/en/latest/rmr.7.html.

[19] Bonati L., Polese M., D’Oro S., Basagni S., Melodia T. (2020). Open, Programmable, and Virtualized 5G Networks: State-of-the-Art and the Road Ahead. Comput. Netw..

[20] Pan Z., Qu Z., Chen Y., Li H., Wang X. (2020). A Distributed Assignment Method for Dynamic Traffic Assignment Using Heterogeneous-Adviser Based Multi-Agent Reinforcement Learning. IEEE Access.

[21] Yang H., Yuan J., Li C., Zhao G., Sun Z., Yao Q., Bao B., Vasilakos A.V., Zhang J. (2022). BrainIoT: Brain-Like Productive Services Provisioning With Federated Learning in Industrial IoT. IEEE Internet Things J..

[22] Elsayed M., Erol-Kantarci M. Reinforcement learning-based joint power and resource allocation for URLLC in 5G. Proceedings of the 2019 IEEE Global Communications Conference, GLOBECOM 2019–Proceedings.

[23] Mnih V., Kavukcuoglu K., Silver D., Graves A., Antonoglou I., Wierstra D., Riedmiller M. (2013). Playing atari with deep reinforcement learning. arXiv.

[24] Yongacoglu B., Arslan G., Yuksel S. Reinforcement Learning for Decentralized Stochastic Control. Proceedings of the 2019 IEEE 58th Conference on Decision and Control (CDC).

